# Integrated Ink Printing Paper Based Self‐Powered Electrochemical Multimodal Biosensing (IFP^−Multi^) with ChatGPT–Bioelectronic Interface for Personalized Healthcare Management

**DOI:** 10.1002/advs.202305962

**Published:** 2023-12-31

**Authors:** Chuanyin Xiong, Weihua Dang, Qi Yang, Qiusheng Zhou, Mengxia Shen, Qiancheng Xiong, Meng An, Xue Jiang, Yonghao Ni, Xianglin Ji

**Affiliations:** ^1^ College of Bioresources Chemical & Materials Engineering Shaanxi University of Science and Technology Xi'an 710021 China; ^2^ School of Chemistry and Materials Engineering Huizhou University Huizhou 516007 China; ^3^ College of Mechanical and Electrical Engineering Shaanxi University of Science and Technology Xi'an 710021 China; ^4^ Department of Chemical and Biomedical Engineering The University of Maine Orono ME 04469 USA; ^5^ Oxford‐CityU Centre for Cerebro‐Cardiovascular Health Engineering (COCHE) City University of Hong Kong Hong Kong Hong Kong SAR 999077 China

**Keywords:** ChatGPT–bioelectronic interface, electrochemical multimodal device, multimodal biosensing, paper based, personal healthcare

## Abstract

Personalized healthcare management is an emerging field that requires the development of environment‐friendly, integrated, and electrochemical multimodal devices. In this study, the concept of integrated paper‐based biosensors (IFP^−Multi^) for personalized healthcare management is introduced. By leveraging ink printing technology and a ChatGPT–bioelectronic interface, these biosensors offer ultrahigh areal‐specific capacitance (74633 mF cm^−2^), excellent mechanical properties, and multifunctional sensing and humidity power generation capabilities. More importantly, the IFP^−Multi^ devices have the potential to simulate deaf‐mute vocalization and can be integrated into wearable sensors to detect muscle contractions and bending motions. Moreover, they also enable monitoring of physiological signals from various body parts, such as the throat, nape, elbow, wrist, and knee, and successfully record sharp and repeatable signals generated by muscle contractions. In addition, the IFP^−Multi^ devices demonstrate self‐powered handwriting sensing and moisture power generation for sweat‐sensing applications. As a proof‐of‐concept, a GPT 3.5 model‐based fine‐tuning and prediction pipeline that utilizes recorded physiological signals through IFP^−Multi^ is showcased, enabling artificial intelligence with multimodal sensing capabilities for personalized healthcare management. This work presents a promising and ecofriendly approach to developing paper‐based electrochemical multimodal devices, paving the way for a new era of healthcare advancements.

## Graphical abstract

A novel concept of integrated paper‐based biosensors (IFP^−Multi^) for personalized healthcare management is proposed. The as‐fabricated biosensors offer ultrahigh areal specific capacitance, excellent mechanical properties, and multifunctional sensing and humidity power generation capabilities. All in all, this work presents a promising and ecofriendly approach to developing paper‐based electrochemical multimodal devices, paving the way for a new era of healthcare advancements.

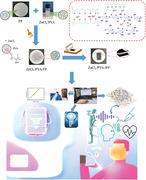

## 1 Introduction

The rapid development of flexible wearable electronics has led to an increasing demand for environmentally friendly, portable, and efficient energy storage systems for personalized healthcare management.^[^ ^1^, ^2^, ^3^, ^4^ ^]^ Integrated, multifunctional, and high‐performance electrochemical multimodal devices^[^ ^5^, ^6^ ^]^ have emerged as a promising solution, with supercapacitors being one of the most important energy storage devices due to their low cost, lightweight, high power density, fast charging and discharging speed, long service life, and good safety. However, the electrolyte and solid electrolyte, critical components of supercapacitors, still affect the energy density of the device to a great extent.^[^ ^7^, ^8^ ^]^ Solid hydrogel electrolytes have the advantages of flexibility, easy packaging, and high safety performance, making them widely used in the assembly of supercapacitors. However, their instability in the environment, including rapid water evaporation and electrolytic salt precipitation, limits their ionic conductivity and energy density. Improving their water retention ability and designing a suitable sandwich structure can improve their ionic conductivity and achieve high energy density^[^ ^9^ ^]^

Currently, paper has become an ideal flexible substrate due to its rough and porous surface, abundant functional groups, as well as its low cost and easy accessibility. Chiang et al.^[^ ^10^, ^11^ ^]^ discussed the deposition of Ag nanoparticles onto different substrates, such as cellulose paper and SBS elastic fibers, resulting in the production of low‐cost, safe, environmentally friendly, flexible wearable sensors. As is known to all, the choice of materials for manufacturing sensors depends on factors such as the sensor's application, availability, and overall manufacturing cost. Nowadays, various nanomaterials can be used as electrode materials for sensors, including various carbon‐based nanomaterials and metal nanoparticles.^[^ ^11^ ^]^

To address these challenges and design high‐performance electrochemical multimodal devices for application in different scenarios, paper has become an ideal flexible substrate due to its rough, porous surface and rich functional groups, as well as its low cost and easy availability.^[^ ^12^, ^13^, ^14^, ^15^, ^16^ ^]^ However, paper is insulated and shows poor mechanical strength and toughness under long‐term mechanical deformation^[^ ^17^ ^]^ Thus, designing a high‐performance paper‐based electrochemical multimodal device with good flexibility and tensile strength is necessary.

In recent years, ZnCl_2_/PVA, as a gel electrolyte, has attracted the attention of researchers due to some advantages of ZnCl_2_/PVA solid gel electrolyte^[^ ^18^ ^]^ Specifically, PVA can disperse well in a solution containing ZnCl_2_. At the same time, zinc ions can form coordination bonds with the hydroxyl groups on PVA, and the interconnected PVA molecular chains intertwine to form a 3D cross‐linked network structure, which is conducive to obtaining good flexibility and mechanical strength, and enhances ion transport rate. Furthermore, due to the water retention ability and appropriate interlayer structure of PVA/ZnCl_2_, combined with the good flexibility and rich porous structure of paper‐based devices, these factors synergistically improve the electrochemical characteristics of the entire paper‐based energy storage device^[^ ^18^ ^]^ In addition, due to the abundance of oxygen‐containing functional groups such as hydroxyl, carboxyl, and carbonyl groups in PVA and cellulose, they can release a large number of protons in humid environments, thereby generating electricity. Therefore, the combination of ZnCl_2_/PVA and paper‐based materials has potential application value for developing flexible multifunctional paper‐based materials.

In this work, we present a novel approach to fabricating integrated paper‐based electrochemical multimodal devices by incorporating ink printing^[^ ^19^ ^]^ technology and a ChatGPT–bioelectronic interface. We introduce ZnCl_2_/PVA into ink printing paper‐based materials to create an integrated paper‐based electrochemical multimodal device^[^ ^16^ ^]^ with ultrahigh areal specific capacitance, excellent mechanical properties, and sensing and humidity power generation multifunctional characteristics. Compared to most nanomaterial‐based sensors, the as‐fabricated sensor not only ensures good sensing characteristics but also offers a more convenient fabrication process with shorter production cycles and lower material costs. It also exhibits certain innovative features as it allows for the free design of sensor shapes, enabling customization for a wider range of applications. In addition, as expected, it can be also used simultaneously as a supercapacitor and humidity generator, making it suitable for various application scenarios. Besides, a more important innovation is that our work can perfectly integrate with a revolutionary technology called ChatGPT, which uses ChatGPT's deep learning technology to analyze and respond to the large amount of text data generated by the aforementioned sensing, opening up new possibilities for personalized medical management.^[^ ^20^, ^21^ ^]^ The ChatGPT–bioelectronic interface enables the device to monitor physiological and biochemical parameters and provide customized advice for personalized healthcare management tailored to their individual needs. Our work presents a promising approach for the development of integrated, multi‐functional, and environment‐friendly paper‐based electrochemical multimodal devices for personalized healthcare management, paving the way for a new era of personalized healthcare management.

## 2 Results and Discussion

### 2.1 Design and Logic of IFP^−Multi^ with ChatGPT–Bioelectronic Interface

**Figure 1:**
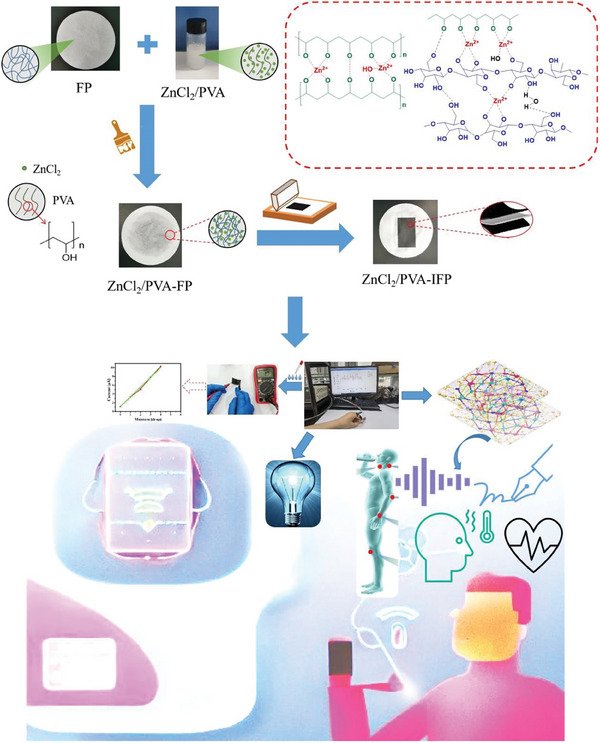
Design and logic of IFP^−Multi^ with ChatGPT‐Bioelectronic Interface. The preparation process and crosslinking mechanism of ZnCl_2_/PVA‐IFP was illustrated, where screen printing was utilized for fabricating ZnCl_2_/PVA‐IFP, with conductive ink transferred to a ZnCl_2_/PVA‐FP substrate and then simply dried in air to print the other side in the same way. The potential applications of the ZnCl_2_/PVA‐IFP hybrid in personalized healthcare management includes voice, handwriting, humidity, sweat, body motion signal acquisition and identification, making it a promising device for the future of healthcare.

To meet the increasing demand for personalized healthcare management, an integrated electrochemical multimodal device (IFP^−Multi^) with high flexibility, power generation potential, and versatility was designed with ZnCl_2_/PVA‐carbon ink filter paper (IFP) (**Figure** **1**). Briefly, the fabrication process of ZnCl_2_/PVA‐IFP starts from screen printing, with conductive ink transferred to a ZnCl_2_/PVA‐FP substrate and then simply dried in air to print the other side in the same way. The resulting integrated electrochemical multimodal device has high areal‐specific capacitance, energy density, and excellent stimulus‐response properties making it suitable for a wide range of applications in personalized healthcare management. IFP^−Multi^ showed excellent sensing characteristics, i.e., detecting physiological signals, handwriting signal recognition, humidity and sweat monitoring. By incorporating ChatGPT‐bioelectronic interface, this device can be used for multimodal biosensing, enabling real‐time monitoring of physiological and biochemical parameters and provide personalized healthcare management advice (Figure 1). This promising approach has the potential to revolutionize personalized healthcare management and promote better health outcomes for individuals.

### 2.2 Morphological and Structural Characterization

**Figure 2:**
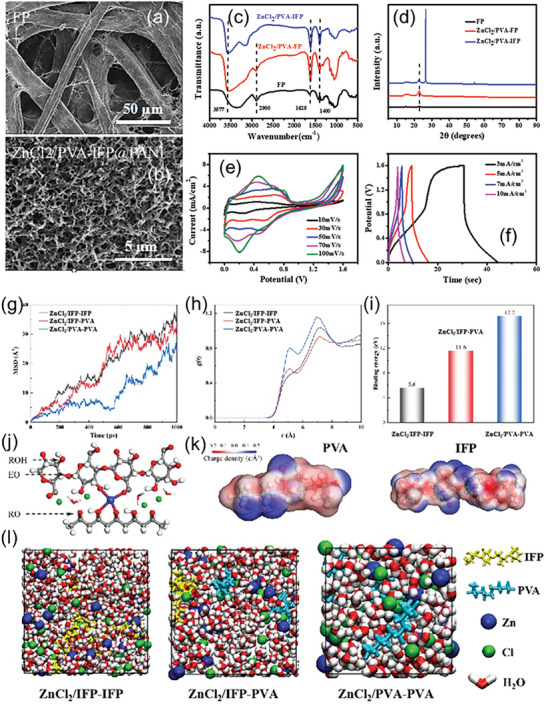
Morphological and structural characterization. Scanning electron microscope (SEM) images of a) filter paper (FP) and b) ZnCl_2_/PVA‐IFP@PANI. The composition of the material was further determined by examining c) the infrared and d) XRD patterns of FP, ZnCl_2_/PVA‐FP, and ZnCl_2_/PVA‐IFP. e,f) The electrochemical properties of ZnCl_2_/PVA‐IFP@PANI were tested at different scan rates and current densities. Zn^2+^ conductivity and transport mechanism in carbonized paper‐based materials. g) Mean square displacement of the Zn^2+^ in different systems. h) Calculated radial distribution function of Zn–O_chain_ in different systems. i) Binding energy of Zn^2+^ to its coordination chains in the different systems. j) Diagram showing the environment of Zn^2+^ in the ZnCl_2_/IFP‐PVA system with different oxygen atom. C, H, O, Zn and Cl atoms are denoted with gray, white, red, blue and green spheres, respectively. k) Charge density distribution for PVA chain and IFP chain, where blue area represents electron loss and red area represents electron enrichment. l) Structural snapshots from MD simulations of ZnCl_2_/IFP‐IFP, ZnCl_2_/IFP‐PVA and ZnCl_2_/PVA‐PVA system. IFP chain and PVA chain are denoted with yellow and cyan, respectively.

Further, we characterize the ZnCl_2_/PVA‐IFP structures with scanning electron microscope (SEM). The multiscale structure of filter paper (FP), ZnCl_2_/PVA‐FP and ZnCl_2_/PVA‐IFP were examined at different magnifications (**Figure** **2**a,b, and Figure S1, Supporting Information). The cross‐linked cellulose fiber networks of FP were observed to overlap and interlace with each other, forming a closely stacked and connected skeleton structure (Figure S1a–c, Supporting Information). The surface of FP was found to be rough, loose, and porous, which provided flexibility to the substrate. SEM images of ZnCl_2_/PVA‐FP (Figure S1d–f, Supporting Information) showed the presence of a cross‐linking structure on the surface of FP due to the hydrogen bonds formed between the hydroxyl of PVA and the oxygen‐containing functional groups, such as hydroxyl, carboxyl, and carbonyl, on the FP surface. Carbon conductive ink was evenly printed on both sides of the PVA/ZnCl_2_@FP using screen printing, which formed a sandwich structure and facilitated ion transmission when the gel electrolyte ZnCl_2_/PVA remained wet at room temperature (Figure S1g–i, Supporting Information). An integrated flexible paper‐based supercapacitor was fabricated without adding additional electrolyte. To improve the electrochemical performance of ZnCl_2_/PVA‐FP, PANI was polymerized on its surface using electrical polymerization. SEM images of ZnCl_2_/PVA‐IFP@PANI (Figure S1j–i, Supporting Information) showed the presence of a nanorod‐like structure of PANI, indicating that it was well‐polymerized on the surface of ZnCl_2_/PVA‐FP.

The composition of the material was further determined by examining the infrared and XRD patterns of FP, ZnCl_2_/PVA‐FP, and ZnCl_2_/PVA‐IFP (Figure 2c,d). The characteristic peaks of FP near 3340 and 2900 cm^−1^ were attributed to the tensile vibration of ─OH and C─H, respectively, which are the main characteristic absorption peaks of cellulose. The stretching vibration peak of hydroxyl group in ZnCl_2_/PVA‐FP appeared at 3577 cm^−1^ due to the formation of hydrogen bond, and the hydroxyl bending vibration occurred at 1400 cm^−1^. The SP_2_ carbon‐carbon stretching vibration was observed at 1625 cm^−1^. Additionally, FP displayed an obvious peak near 23°, which is the characteristic diffraction peak of cellulose. There was also a sharp, strong peak near 26°, corresponding to the crystal face of graphite. These findings provide a comprehensive understanding of the material composition and structure of the ZnCl_2_/PVA‐IFP hybrid, which demonstrates its potential for various applications in the field of personalized healthcare management. An integrated IFP^−Multi^ was prepared by screen printing, in which a FP substrate impregnated with ZnCl_2_/PVA served as a gel electrolyte and diaphragm for direct electrochemical testing. To improve the low power density of conductive ink as a carbon material, the conductive polymer PANI was introduced to further fabricate ZnCl_2_/PVA‐IFP@PANI. The synergistic effect of the two materials led to improved comprehensive performance of the supercapacitor. The electrochemical properties of ZnCl_2_/PVA‐IFP and ZnCl_2_/PVA‐IFP@PANI were tested and compared (Figure 2e,f, and Figures S2 and S3, Supporting Information). These excellent electrochemical properties can be compared with some excellent work in the past, as displayed in Table S1 (Supporting Information).

### 2.3 Molecular Dynamics Simulation

Molecular dynamics (MD) simulation^[^ ^22^ ^]^ is a powerful tool for investigating Zn^2+^ conductivity and transport mechanisms in three systems, namely ZnCl_2_/IFP‐IFP, ZnCl_2_/IFP‐PVA, and ZnCl_2_/PVA‐PVA (Figure 2g–l). Compared with others’ work, it is worth noting that as mentioned above, ZnCl_2_/PVA has good water retention capacity and appropriate interlayer structures, which is beneficial for improving ion conductivity of the whole paper‐based material. Moreover, zinc ions form coordination bonds with the hydroxyl groups on PVA, and the interconnected PVA molecular chains intertwine to form a 3D cross‐linked network structure, which enhances ion transport rate. In addition, in the ZnCl_2_/PVA‐IFP hybrid, paper‐based serves as both a flexible substrate for the device and as a separator for the supercapacitor, thus constructing a membrane‐free integrated supercapacitor. All factors endow the ZnCl_2_/PVA‐IFP paper‐based composites with better energy storage characteristics. The mean square displacement (MSD) of Zn^2+^ in each system is shown in Figure 2g, where lower values of MSD indicate slower diffusion. The pure ZnCl_2_/PVA‐PVA system exhibits the slowest Zn^2+^ diffusion, while the pure ZnCl_2_/IFP‐IFP system shows the fastest Zn^2+^ diffusion. The Zn^2+^ diffusion is enhanced as the IFP and PVA chains are mixed in the ZnCl_2_/IFP‐PVA system. The radial distribution functions (RDF) of Zn^2+^ and O atoms of chains were calculated to describe the interaction between them (Figure 2h). A stronger binding effect is observed between chains and Zn^2+^ in the ZnCl_2_/IFP‐PVA system, as indicated by the increased peak height at *r* = 5.1Å. The binding energy of Zn^2+^ in the ZnCl_2_/IFP‐PVA system is 11.6 eV, which is much larger than that in the ZnCl_2_/IFP‐IFP system (5.6 eV) (Figure 2i). However, the strong Zn‐O chain bonding in the ZnCl_2_/IFP‐PVA system also prevents Zn^2+^ diffusion. Zn^2+^ can form multiple coordinations with rich oxygen functional groups, including hydroxyl (ROH), alkoxide (RO─), ether (EO) moieties, water molecules, and Cl─ (Figure 2j). The charge density distribution of PVA and IFP chains was calculated and plotted to clarify the effect of each part on Zn^2+^ (Figure 2k). More and darker red areas are observed on the IFP chain, indicating its weak binding effect on Zn^2+^. The theoretical calculations suggest that the mix of IFP and PVA chains not only enhances the structural strength through strong coordination of Zn^2+^ in the ZnCl2/IFP‐PVA system, but also makes the conduction more stable by restricting Zn^2+^ diffusion.

### 2.4 Multimodal Biosensing: Self Powered Handwriting Sensor

To evaluate the adaptability of the ZnCl_2_/PVA‐FP composites to external forces, a tensile test was conducted and shown in Figure S4a (Supporting Information). The shape variable of the FP sample was 2.437% at a stress of 6.3 MPa, indicating low toughness and vulnerability to destruction during use. However, when ZnCl_2_/PVA was added to the FP, the shape variable of the resulting composite was 18.722% at a stress of 6.037 MPa, which was 16% higher than that of the pure FP sample at the same strength. This suggests that ZnCl_2_/PVA‐FP composites have more application potential in flexible devices. The IFP‐Multi composite obtained after screen printing exhibited even better strength and toughness, with a shape variable of 21.062% at 6.8 MPa. Based on the excellent tensile properties of the flexible IFP^−Multi^, a simple electrical test was conducted. Bending the IFP^−Multi^ from 0 to 180° produced a corresponding current signal, which was stable and sensitive to continuous changes in action. This feature enables the flexible IFP‐Multi to respond quickly to different human movements in real time, making it a promising sensor for advanced body motion monitoring (Figure S4b, Supporting Information).

**Figure 3:**
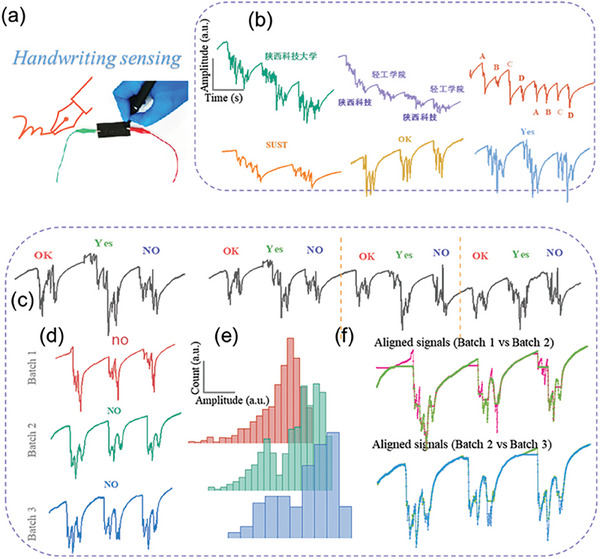
Multimodal biosensing: Self powered handwriting sensor. a) Illustration and photograph of handwriting signal acquisition. b) Representative handwriting signals of various Chinese or English phrases. c) Repetitive handwriting tests of similar phrases. d) Capital and lower‐case letter handwriting signal demonstration. e) Histogram of the amplitude distribution of the signal patterns in (d). f) Dynamic time warping aligned distance among the signal patterns in (d).

Besides, a series of tests on IFP^−Multi^ sensors in the field of self‐powered tactile sensors were also conducted. As shown in **Figure** **3**a, two sections of ZnCl_2_/PVA‐FP were connected to both ends of the electrochemical comprehensive tester. The experimenter can generate special peak shape and number of current output signals according to the different pressure of the pen tip by writing characters on the surface with a ball pen, so as to explore the application of IFP^−Multi^ sensor in the high‐sensitivity handwriting recognition system. First, the experimenter wrote Chinese characters on the surface of IFP^−Multi^ sensor, including “Shaanxi University of Science and Technology”, “Shaanxi science and technology” and “Light Industry College.” The corresponding sensing maps were shown in Figure 3a,b. It can be seen that under the same characters, although the writing pressure varies with the writing time and the current output signal varies in detail, the curves still have obvious specificity, indicating that the IFP^−Multi^ sensor has good recognition in current writing patterns. It can be found that the output current corresponding to the same text is repeatable, while the output current corresponding to different texts is significantly different. Therefore, the IFP^−Multi^ has high sensitivity, recognition and repeatability when used as a self‐powered tactile sensor. The sensing inputs has been repeatedly written for three times. The three times of characters have similar current output signals, indicating that the IFP^−Multi^ sensor has good repeatability (Figure 3c). In addition, Figure 3d–f shows the sensing curves of “no” and “NO” for three consecutive times of writing. The difference in the case of words will lead to the difference in the pen tip pressure, which will lead to the difference in the output current signal. It shows that the IFP^−Multi^ has good specificity. Moreover, when writing the same text, the instability of the pen tip pressure caused by handwriting will lead to the subtle difference of the output current signal, which indicates that it has high sensitivity. The amplitude histogram (Figure 3e) and the dynamic time warping aligned distance^[^ ^23^ ^]^ (Figure 3f) also indicate the similar trend. All in all, the above test results show that the IFP^−Multi^ has high sensitivity, recognition, and repeatability in the application of self‐powered tactile sensors. Besides, the IFP^−Multi^ also shows excellent sensitivity in pressure and capacitance sensing (Videos S1–S4, Supporting Information), demonstrating potential applications in these fields.

### 2.5 Multimodal Biosensing: Moisture Power Generation for Sweat Sensing

**Figure 4:**
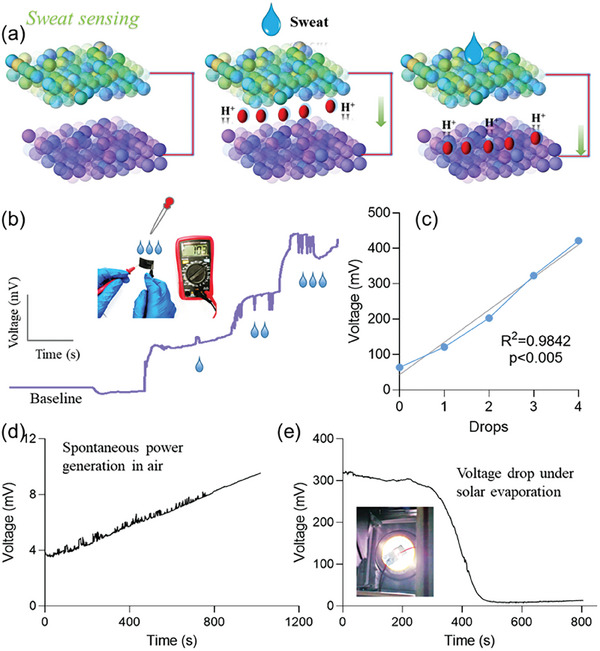
Multimodal biosensing: Moisture power generation for sweat sensing. a) The mechanism, by which moisture generates electricity based on the ZnCl_2_/PVA‐IFP. b) The power generation process of ZnCl_2_/PVA‐IFP composite in artificially assisted humidity environment. c) The relationship of ZnCl_2_/PVA‐IFP between moisture and generating capacity. d) Spontaneous electric process of ZnCl_2_/PVA‐IFP composite in air. e) Voltage drop of ZnCl_2_/PVA‐IFP composite under a solar evaporation.

The current and voltage test process of IFP^−Multi^ under different humidity power generation conditions (**Figure** **4**a) was performed by connecting the two sides of IFP^−Multi^ with a multimeter probe to detect the current and voltage signals under different environmental humidity conditions. For more intuitive details about the testing process, please refer to Figure S5 in the Supporting Information.

Subsequently, after adding one drop, two drops and three drops of distilled water drop by drop with a rubber tipped dropper, it was found that with the action of dropping water drops, the voltage showed a sudden rise. However, after adding the third drop, the voltage reached about 330 mV (Figure 4b). This was because the concentration difference of H^+^ increased, resulting in a larger potential difference.

Obviously, in the dry environment without dripping water, due to the formation of coordination bonds and hydrogen bonds within the ZnCl_2_/PVA‐IFP composite material, the intermolecular force increases, and 1.1 µA weak current signal and 64 mV voltage signal were obtained, respectively. Subsequently, when a drop of water was added, 3.3 µA current and 121 mV voltage were produced, respectively. When two drops of water were added, 5.1 µA current and a voltage of 203 mV were obtained, respectively. When three drops of water were added, 7.8 µA current and 323 mV voltage were obtained, respectively. When four drops of water were added, 10.3 µA and a voltage of 422 mV were produced, respectively (Figure 4c). That is to say, when water was added drop by drop, the current and voltage gradually increase with the increase of humidity. With the drop by drop increase of water droplets, the micro current and voltage generated gradually increase, and the slope is highly consistent (*R* ^2^ = 0.9842), indicating that there is a good linear relationship between power generation and water content.

Through the above simple humidity power generation test, it was found that the IFP^−Multi^ had potential humidity power generation function, and then its humidity power generation mechanism was studied. Specifically, First, Figure 4d shows the whole process of humidity power generation of IFP^−Multi^ under the air environment. This is because the air contains water molecules, and the material can absorb the water molecules in the air, thus generating a difference in H^+^ concentration, so that it can perform self‐generating function. Then, the ZnCl_2_/PVA‐IFP composite material with water drops was irradiated under a solar light intensity (Figure 4e). It was found that with the increase of time, the water gradually evaporated, and the voltage of IFP^−Multi^ also gradually decreased. When the water evaporated, the voltage at both ends returned to the initial value.

To sum up, the IFP^−Multi^ device can also be used as an effective test instrument for detecting the change of environmental humidity. This may be because PVA, H_2_O and cellulose of IFP^−Multi^ contain a large number of hydrophilic groups, such as hydroxyl groups, which can decompose and release protons in a large amount under wet conditions. When no additional water is added to IFP^−Multi^, the coordination bond between Zn^2+^ and hydroxyl group and the weak hydrogen bond between hydroxyl group and hydroxyl group improve the intermolecular force. Meanwhile, the existence of the double conductive ink layer has a certain water retention effect^[^ ^24^ ^]^ so there are still weak current and voltage signals. When water is added to the surface of IFP^−Multi^ composite, hydrogen bonds are formed due to the presence of hydroxyl groups in water and IFP^−Multi^, and the concentration of H^+^ in water is much higher than that of IFP^−Multi^ composite. Therefore, the current is generated by the movement of H^+^ from the high concentration region to the low concentration region, and the voltage is also generated.

### 2.6 Multimodal Biosensing: Self‐Powered Physiological Signal Monitoring

**Figure 5:**
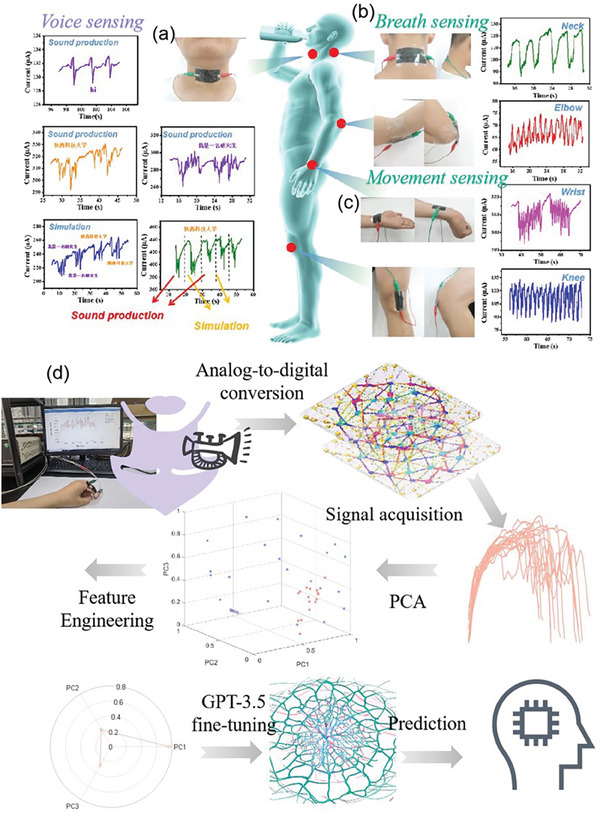
Multimodal biosensing: Self‐powered physiological signal monitoring. The response of IFP^−Multi^ to a) laryngeal sound changes, b) breath, c) body movement. d) A pipeline for GPT 3.5 based model fine‐tuning and prediction with our recorded physiological signals.

The IFP^−Multi^ can also be used to simulate deaf‐mute vocalization. First of all, as shown in **Figure** **5**a, the response of IFP‐Multi to laryngeal sound changes is stable and sensitive by uttering a simple English word “Hi”. It can be seen that the corresponding current signals obtained by saying “Hi” three times are very similar, which proves that the response of IFP^−Multi^ to laryngeal sound changes is stable and sensitive even for simple words. Therefore, the response of IFP^−Multi^ to relatively long Chinese phrases was further tested. Through normal speech and simulated speech, by saying “Shaanxi University of Science and Technology” and “I am a graduate student” (In Chinese), it can be seen that the same words will have similar electrical signals, which can be highly repeated, and different words will have significantly different electrical signals, proving that the response of the change of voice in the throat is stable and sensitive. Furthermore, to the best of our knowledge, deaf and mute people cannot completely reveal every syllable when making sound, resulting in ambiguous syllables, mainly through the vibration of the throat, mouth or sign language to express ideas. Based on this, the voice of the deaf and mute is simulated. The syllables “Shaanxi University of Science and Technology” and “I am a graduate student” (In Chinese) are vibrated through the throat. It can also be intuitively seen that the same words will have similar electrical signals, and different words will have significantly different electrical signals. Then, through the signal strain detection and comparison between normal speech and simulated speech, it can be found that the two still have similar electrical signals even if the speed of speech or simulation is different. Based on this, it can be considered that using IFP^−Multi^, a set of speech tools for deaf people can be programmed to facilitate their life.

To study the application of IFP^−Multi^ in wearable sensor, as shown in Figure 5b,c, both ends of IFP^−Multi^ were fixed on the throat, nape, elbow, wrist, and knee of the subject with adhesive tape for strain sensing test. The results showed that the sensor could detect sharp and repeatable signals generated by muscle contractions in the back of the neck during a simple nod. When the sensor is connected to the elbow and wrist, it can detect electrical signals generated by bending motion, and then connected to the knee to detect sensitive electrical output through simple walking. IFP^−Multi^ has sensitive and highly repeatable current output, probably because the PVA in IFP^−Multi^ contains more hydroxyl groups. Therefore, when the self‐assembled sensor is in contact with human skin, the PVA permeability is poor, leading to skin sweating. This allows the skin to bind more tightly to the flexible IFP^−Multi^, resulting in hypersensitivity to pressure.

### 2.7 ChatGPT–Bioelectronic Interface

Furthermore, as a proof‐of‐concept study, here we demonstrate a pipeline for GPT 3.5 based model^[^ ^25^ ^]^ fine‐tuning and prediction with our recorded physiological signals (Figure 5d). For starter, our biosensor interface pipeline involves acquiring voltage or current signals using our ink printed electrochemical multimodal device. Here, the changes in electrical signals were detected by directly connecting the sensor components to the electrochemical workstation. Furthermore, these signals are then subjected to analog‐to‐digital conversion, allowing for more precise signal processing. In the second step, we remove the baseline of the recorded signals, which eliminates the effect of any drifts and makes the signals comparable to each other. Next, we segment the signals into single words using an automated approach (Figure S6, Supporting Information), which allows for more efficient processing in later steps. To further reduce the dimensionality of the data, we employ principal component analysis (PCA)^[^ ^26^ ^]^ in the third step. This technique allows us to identify the most important variables in the data and to reduce the number of variables to a manageable number. We found that using only three vectors was sufficient to explain 95.7% (Figure S7, Supporting Information) of the variance in the data. In the fourth step, we use these three vectors to fine‐tune the GPT 3.5 language model. By training the model on this reduced dataset, we can achieve more accurate predictions of biosensor data. We use several rounds of conversation to refine the model and optimize its performance. Finally, in the fifth step, we use the fine‐tuned GPT 3.5 model for prediction. This model has been optimized to recognize patterns in biosensor data and can accurately predict other untrained data points. Our biosensor interface pipeline appears a potential as a powerful tool for analyzing and predicting biosensor data, leveraging advanced signal processing and language modeling techniques to achieve high levels of accuracy and efficiency.

**Figure 6:**
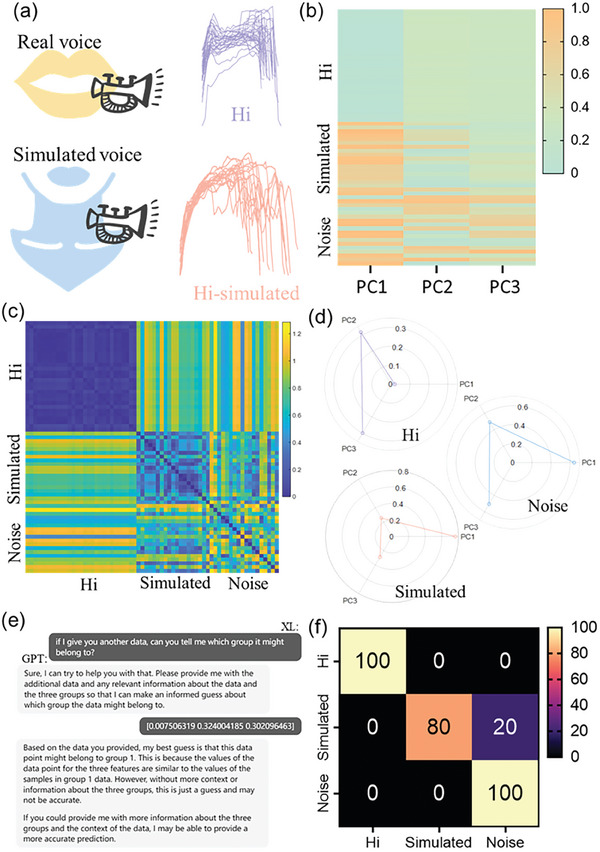
ChatGPT–bioelectronic interface. a) Extracted and pooled “Hi” real voice signals (29 recordings) and simulated signals (17 recordings). b) Feature heatmap of real voice signals, simulated signals and introduced noise signals. c) Intra and inter group similarity of voice, simulated and introduced noise signals. d) Radar plot of the averaged feature distribution. e) ChatGPT fine tuning interface. f) Confusion matrix of the ChatGPT prediction accuracy.

To the best of our knowledge, deaf and mute people cannot completely reveal every syllable when making sound,^[^ ^27^, ^28^ ^]^ resulting in ambiguous syllables, mainly through the vibration of the throat, mouth or sign language to express ideas. Based on this, the voice of the deaf and mute is simulated. Therefore, after the construction of the GPT‐bioelectronic interface, we wonder if our AI powered smart multimodal device can be used to be integrated into the real voice and simulated voice recognition. Hence, we extracted and pooled “Hi” real voice signals (29 recordings) and simulated signals (17 recordings) (**Figure** **6**a and Table S2, Supporting Information). To further test the robustness of our model, we also include 20 noise signals as interference (Figure 6b and Figure S8, Supporting Information). The “Hi” real voice group signals and simulated group signals both appear high intra‐group similarity and whereas the noise group signals show quite low intra‐group similarity, indicating our recorded signals have good repeatability and stability (Figure 6c). Radar plots were also demonstrated to show the average feature vector distribution. Unlike the real signal or simulated signals with unique pattern, the noise signal shows a relatively uniform distribution in the three‐vector space due to its noise nature (Figure 6d). As a proof‐of‐concept demonstration, we used 15 samples for training GPT 3.5 model with ≈3‐5 rounds conversation (Figure 6e and Figure S9, Supporting Information). Then we used another batch 15 samples to test the prediction accuracy. It was very exciting to see that the GPT 3.5 model only wrongly identify one samples among the 15 test signals. It was worth to say that with only 15 samples as training group and only ≈3‐5 rounds conversations, the GPT model already achieve a quite good performance (Figure 6f).

## 3 Discussion and Conclusion

Our study has successfully developed a simple and effective method to construct ultra strong and multifunctional paper‐based supercapacitors with superior super capacitive behavior, sensing, and humidity power generation characteristics. The integrated paper‐based supercapacitors demonstrate excellent mechanical properties due to the interaction between nano‐fiber cellulose and PVA/ZnCl_2_, while PVA/ZnCl_2_ acts as a gel electrolyte, resulting in ultrahigh areal specific capacitance and specific energy density. Theoretical calculations indicate that the mixture of IFP chains and PVA chains enhances structural strength via strong coordination of Zn^2+^ in the ZnCl_2_/IFP‐PVA system, while also providing stable conduction by restricting Zn^2+^ diffusion. This research demonstrates the potential application of integrated paper‐based supercapacitors in the fields of human body sensing, humidity power generation, and photothermal conversion. Our work provides a new idea and reference for the development, application, and recycling of green biomass materials.

In addition, our study presents a promising approach for personalized healthcare management through the creation of an integrated electrochemical multimodal device with high flexibility, power generation potential, and versatility. We believe that leveraging the structural shape of soft materials allows for the design of sensor devices with diverse structures and shapes, such as bracelets, collars, and more.^[^ ^14^, ^29^, ^30^, ^31^, ^32^ ^]^ By integrating the sensor components with soft materials in a tailored manner, we can further enhance the sensitivity and stability of the sensors. The IFP^−Mult^ **^i^** device demonstrated excellent electrochemical performance, with high areal specific capacitance and energy density. This device has the potential to be used for a wide range of applications in personalized healthcare management, including as a sensor for detecting physiological signals of human movement, a self‐powered tactile sensor and handwriting recognition system, and for multimodal biosensing with the incorporation of ChatGPT‐bioelectronic interface. The incorporation of the ChatGPT‐bioelectronic interface enables multimodal biosensing, further expanding the potential applications of this device. The IFP^−Multi^ device also has the potential to be used for detecting environmental humidity changes or for sweat monitoring, making it a versatile device for use in a variety of settings.

To facilitate the analysis and prediction of biosensor data, we developed a biosensor interface pipeline that combines advanced signal processing and language modeling techniques. GPT 3.5, based on the GPT‐3 architecture, represents a significant advancement in natural language processing and language generation capabilities. It has demonstrated remarkable performance in various language‐related tasks, including text completion, translation, summarization, and dialogue generation. By utilizing GPT 3.5, we leverage its state‐of‐the‐art language modeling capabilities to predict physiological signals accurately. The integration of ChatGPT, which is a variant of the GPT model specifically designed for interactive conversations, serves as a vital component in our research. The ChatGPT fine‐tuning interface allows us to enable artificial intelligence with multimodal sensing capabilities for personalized healthcare management. By fine‐tuning the GPT 3.5 language model on a reduced dataset, we were able to achieve more accurate predictions of biosensor data, which can have potential applications in speech recognition and other multimodal sensing applications. Our AI‐powered multimodal device showed potential in voice recognition applications for deaf and mute individuals, demonstrating the versatility and potential of our technology. Future studies could further optimize our pipeline and explore its potential applications in other areas of biosensor data analysis and prediction.

Overall, our integrated paper‐based supercapacitors and electrochemical multimodal device with high flexibility, power generation potential, and versatility have great potential for practical applications in various fields, and further optimization of our approach could lead to even better performance and wider applications.

## 4 Experimental Section

### Materials

Sulfuric acid is purchased from Sinopharm Chemical Reagent Co. Ltd., China. Aniline (AN) is supplied by Macklin Reagent Co., Ltd., China. PVA and ZnCl_2_ are supplied by Damao Reagent Co., Ltd., China. All the reagents are of analytical grade and used without purification. Qualitative filter paper of Newstar brand is adopted. Conductive printing ink is used from Jiecheng brand Screen Printing ink.

### Preparation of ZnCl_2_/PVA‐FP

By brushing ZnCl_2_/PVA on the surface of the filter paper, the electrolyte can penetrate into the filter paper pores. Because ZnCl_2_/PVA can stay wet for a long time at room temperature, which is conducive to ion flow, it can form the electrolyte and separator part of the integrated device.

### Preparation of ZnCl_2_/PVA‐IFP Supercapacitor

Figure 1 illustrates the process of fabricating ZnCl_2_/PVA‐IFP via screen printing. Conductive ink was first placed on a screen plate (200 mesh), then applied with a hand scraper, the ink was transferred to a ZnCl_2_/PVA‐FP substrate and then simply dried in air to print the other side in the same way. Finally, integrated ZnCl_2_/PVA‐IFP supercapacitor is obtained.

### Preparation of ZnCl_2_/PVA‐IFP@PANI Supercapacitor

Through the above experiments, integrated ZnCl_2_/PVA‐IFP supercapacitor with a length of 30 mm and a width of 20 mm was obtained. In order to further improve the electrochemical performance of supercapacitors, square nickel foam strips and ZnCl_2_/PVA‐IFP strips were connected to the cathode and anode of the stabilized power supply, and placed in 400 mL 0.2 mol L^−1^ aniline and 0.5 mol L^−1^ H_2_SO_4_ aqueous solution, respectively. By controlling the time and adjusting the voltage of the regulated power supply, PANI was deposited on ZnCl_2_/PVA‐IFP, and finally ZnCl_2_/PVA‐IFP@PANI supercapacitor was obtained.

### Characterization

The morphology and structure of the samples were characterized by scanning electron microscope (VEGA 3 S‐4800, HITACHI). X‐ray diffraction (XRD) patterns of these samples were collected with X‐ray diffractometer (D8 Advance, Bruker Germany), using Cu Kα radiation with a scanning rate of 6° min^−1^ from 5° to 90°. Fourier transform infrared spectroscopy (FT‐IR, VERTEX 70, Bruker Optics Corporation; Germany) was carried out to study structure and interface combination of materials. The mechanical properties of the material were tested using servo material multifunctional high and low temperature control testing machine (Gottweil Co., LTD.).

### Electrochemical Characterization

The electrochemical properties of integrated ZnCl_2_/PVA‐IFP and ZnCl_2_/PVA‐IFP@PANI supercapacitors were studied by CHI760E Electrochemical Workstation (Chenhua, Shanghai). Since the material itself has an electrolyte and a diaphragm, no further assembly is required and the sample is tested directly on the working electrode and the counter electrode. Cyclic voltammetry (CV) measurements were carried out at speeds ranging from 10 to 100 mV s^−1^, and galvanostatic charge–discharge (GCD) tests were performed at current densities of 3, 5 and 7 mA cm^−2^, respectively. The specific capacitance *C*_s_, energy density *W* and power density *P* can be calculated from the CV curve as follows^[^ ^33^ ^]^:

(1) $$Cs=\frac{\int_{v_{1}}^{v_{2}}\left|I\right|dv}{Sv\Delta V}$$

(2) $$W=\frac{Cs\Delta v^{2}}{2}$$

(3) $$P=\frac{W}{\Delta t}$$

where *I* is the charging discharge current, *S* is the area of the entire supercapacitor, △*t* is the discharge time, *V* is the scanning rate in the measurement process, and △*V* is the potential window in CV measurement process.

### Human Signal Detection Experiment

To further explore the application of ZnCl_2_/PVA‐IFP in wearable sensors, the electrochemical synthesis tester (P4000+, Princeton, USA) was used to test the signal transmission of the material to detect body movement. Volunteers (a 25 year old male with no physical history) were tested by using VHB tape to attach the ZnCl_2_/PVA‐IFP to various parts of the body, including the nape of the neck, elbow, wrist, knee and throat, to detect physiological signals and to simulate deaf‐mute vocalisms. Subsequently, the application of ballpoint pen to high sensitivity handwriting recognition system was tested by writing on the surface of ZnCl_2_/PVA‐IFP.

### ZnCl_2_/PVA‐IFP Moisture Power Generation Experiment

The multimeter probe was placed on both sides of the integrated ZnCl_2_/PVA‐IFP supercapacitor (40 mm × 20 mm), and the moisture production capacity of the integrated ZnCl_2_/PVA‐IFP supercapacitor was tested by adding water drops on its surface (water drops are the humidity environmental conditions). Then, record current and voltage signals with a digital multimeter (UT33B+, LINI‐T, China). Different writing tests have been repeated 20 times each (subject to change depending on the situation).

### Molecular Dynamics (MD) Simulation

All molecular dynamics (MD) simulations were performed implemented by the largescale atomic/molecular massively parallel simulator (LAMMPS) package^[^ ^34^ ^]^ LigParGen web‐based service^[^ ^35^ ^]^ was used to generate initial structure of FP chain and PVA chain, and obtain the OPLS force‐field parameters and partial atomic charges. The TIP4P water model was adopted in the simulations^[^ ^36^ ^]^ The nonbonded interactions among ions, water molecules and chains described by Lennard‐Jones 12–6 atomistic potential. Initially, the systems with the same mass ratio of synthesis of FP‐PVA@ZnCl_2_ mentioned above were placed in a simulation cell which size depends on maintaining the density of water in the system at 1 g cm^−3^ using PACKMOL.^[^ ^37^ ^]^ Three systems are shown in Figure 1g‐i. Periodic boundary conditions in three dimensions were applied. The cutoff radius for long‐range energy calculations was set to 12 Å. The contribution of long‐range interaction was calculated by particle‐particle‐particle‐mesh (PPPM) solver.^[^ ^38^ ^]^ The Newton's equations of motion were time‐integrated with a time‐step of 0.1 fs. The visual molecular dynamics (VMD)^[^ ^39^ ^]^ was used to visualize the trajectories generated during MD simulations. Each sample was equilibrated via the use of NPT simulations at 298 K and 1 atm over a period of 1 ns. Following this, a further 1 ns simulation was performed in the NVT ensemble. Mean square displacement (MSD) can be computed from the following expression,

(4) $$\mathrm{MSD}=\langle \frac{1}{N}\sum_{i=1}^{N}{\left|r_{d}-r_{d}\left(t_{0}\right)\right|}^{2}\rangle$$

where *N* is the number of equivalent particles the MSD is calculated over, *r* is their coordinations and *d* is the desired dimensionality of MSD. And the radial distribution function (RDF) is calculated by

(5) $$g\left(r\right)=\frac{n\left(r\right)}{\rho 4\pi r^{2}\Delta r}$$

where *n*(*r*) is the average number of particles in a spherical shell with a width of Δ*r* at a distance *r* from the center reference particle, *ρ* is the average density of particles in the system. The binding energy are calculated by

(6) $$E_{\mathrm{binding}}=E_{\mathrm{total}}-\left(E_{\mathrm{chains}}+E_{Zn^{2+}}\right)$$

where *E*_total_ is the energy after stabilization of a system comprising two chains and one Zn^2+^, *E*_chains_ and $E_{Zn^{2+}}$ represent the energy after stabilization of tow chains and one Zn^2+^, respectively. The charge density of each chain was calculated based on the density functional theory using Vienna Ab initio Simulation Package (VASP)^[^ ^40^, ^41^ ^]^ along with the projector augmented wave (PAW) pseudopotentials. The Becke‐Lee‐Yang‐Parr (BLYP) version of the generalized gradient approximation (GGA)^[^ ^42^ ^]^ is used for the exchange‐correction function. The convergence criteria for the total energy and ionic force were 1 × 10^−8^ eV and 0.02 eV Å^−1^, respectively. The cutoff energy of the plane‐wave was set at 400 eV.

### Data Acquisition and Preprocessing

To remove the baseline trend from the signal, a polynomial curve was fitted to the signal using the polyfit function in MATLAB. A second‐degree polynomial was chosen for the curve fitting, and the resulting curve was subtracted from the original signal using the polyval function. The detrended signal was then plotted using the plot function. To segment the signal into individual speech events, the findpeaks function was used to detect peaks in the negative of the detrended signal. The peaks were separated by a minimum peak distance of 30 samples. The resulting peaks were plotted over the detrended signal using the scatter function. The signal was then segmented into individual speech events by extracting the signal between each pair of detected peaks. The segmented signals were analyzed to determine the number of nonzero elements in each segment. Segments with more than 30 nonzero elements were retained for further analysis, while those with fewer than 30 were discarded. The segmented signals were visualized using the plot function. Each speech event was plotted as a separate line. The resulting plot was saved as a figure and displayed without axis labels using the axis off function.

### Dimensionality Reduction and Feature Extraction

Principal component analysis (PCA) was used to reduce the dimensionality of the signal data and extract relevant features. The pca function in MATLAB was used to calculate the principal component coefficients, scores, latent variables, and explained variance. The first three principal components, which explained the majority of the variability in the data, were retained for further analysis. The resulting features were normalized to the range of 0 to 1 using the min–max scaling method. In order to test the robustness of the clustering algorithm to noise, 20 sets of random data were generated and added to the feature matrix as a new class of signals. These random signals were normalized to the same range as the original signals and labeled with a value of 3. The Poe app utilized a ChatGPT chatbot based on the GPT 3.5 architecture for both fine‐tuning and prediction.

### Distance Matrix Calculation

The Euclidean distance between all pairs of signals in the feature matrix was calculated using the pdist2 function in MATLAB. The resulting distance matrix was visualized using the imagesc function. The normalized feature matrix was also visualized using imagesc to show the distribution of the feature values across all signals.

### Radar Plot

To visualize the differences between the three classes of signals, radar plot was generated for each class using the mean values of the first three principal components. The polarplot function in MATLAB was used to plot the mean values on a circular axis with three spokes representing the three principal components.

### Histogram Analysis

To visualize the distribution of the time series data in each batch, a histogram was generated for each matrix using the histogram function in MATLAB.

### Dynamic Time Warping (DTW) Analysis

To compare the similarity between time series data from different batches, the DTW algorithm was used. The dtw function in MATLAB was used to calculate the DTW distance between the time series data in each pair of batches.

## Conflict of Interest

The authors declare no conflict of interest.

## Author Contributions

C.X. conceived the project and supervised the study. W.D. designed, prepared and tested the electrochemical performance of the paper‐based material. Q.Y. helped with optimized the paper‐based material fabrication process. Q.Z. helped with the electrochemical analysis of the paper‐based material. M.S. helped with testing the electrochemical performance of the paper‐based material. Q.X. performed the related component analysis of the paper‐based material. M.A. performed molecular dynamics simulations. X.J. helped with different aspects of the experiments. Y.N. helped with reviewing the paper. X.J. designed the ChatGPT model and reviewed the manuscript.

## Supporting information

Supporting Information

## Data Availability

The data that support the findings of this study are available from the corresponding author upon reasonable request.
