# Supplementary material for: Integrated Ink Printing Paper Based Self‐Powered Electrochemical Multimodal Biosensing (IFP−Multi) with ChatGPT–Bioelectronic Interface for Personalized Healthcare Management
Source: Adv Sci (Weinh). 2023 Dec 31;11(11):2305962. doi: 10.1002/advs.202305962 (PMC10953564; doi:10.1002/advs.202305962)
Supplement: Supplementary file 1 — Supporting Information [file ADVS-11-2305962-s001.pdf]

ADVANCED SCIENCE
Open Access

Supporting Information

for *Adv. Sci.*, DOI 10.1002/advs.202305962

Integrated Ink Printing Paper Based Self-Powered Electrochemical Multimodal Biosensing (IFP^{-Multi}) with ChatGPT–Bioelectronic Interface for Personalized Healthcare Management

Chuanyin Xiong, Weihua Dang, Qi Yang, Qiusheng Zhou, Mengxia Shen, Qiancheng Xiong, Meng An*, Xue Jiang, Yonghao Ni and Xianglin Ji**

Supporting Information

Integrated Ink Printing Paper Based Self-Powered Electrochemical Multimodal Biosensing (IFP^{-Multi}) with ChatGPT-Bioelectronic Interface for Personalized Healthcare Management

Chuanyin Xiong[1]*, Weihua Dang[1], Qi Yang[1], Qiusheng Zhou[1], Mengxia Shen[1], Qiancheng Xiong[2], Meng An[3]*, Xue Jiang[1], Yonghao Ni [4], Xianglin Ji[5]*

[1]College of Bioresources Chemical & Materials Engineering, Shaanxi University of Science and Technology, Xi’an 710021, China
[2]School of Chemistry and Materials Engineering, Huizhou University, Huizhou 516007, China
[3]College of Mechanical and Electrical Engineering, Shaanxi University of Science and Technology, Xi’an 710021, China
[4]Department of Chemical and Biomedical Engineering, The University of Maine, Orono, Maine, 04469, USA
[5]Oxford-CityU Centre for Cerebro-Cardiovascular Health Engineering (COCHE), City University of Hong Kong, Hong Kong SAR, 999077, China

*Correspondence should be addressed to
Prof. Chuanyin Xiong (xiongchuanyin@sust.edu.cn),
Dr. Meng An (anmeng@sust.edu.cn),
Dr. Xianglin Ji (xiangliji3-c@my.cityu.edu.hk)

Supplementary Figures

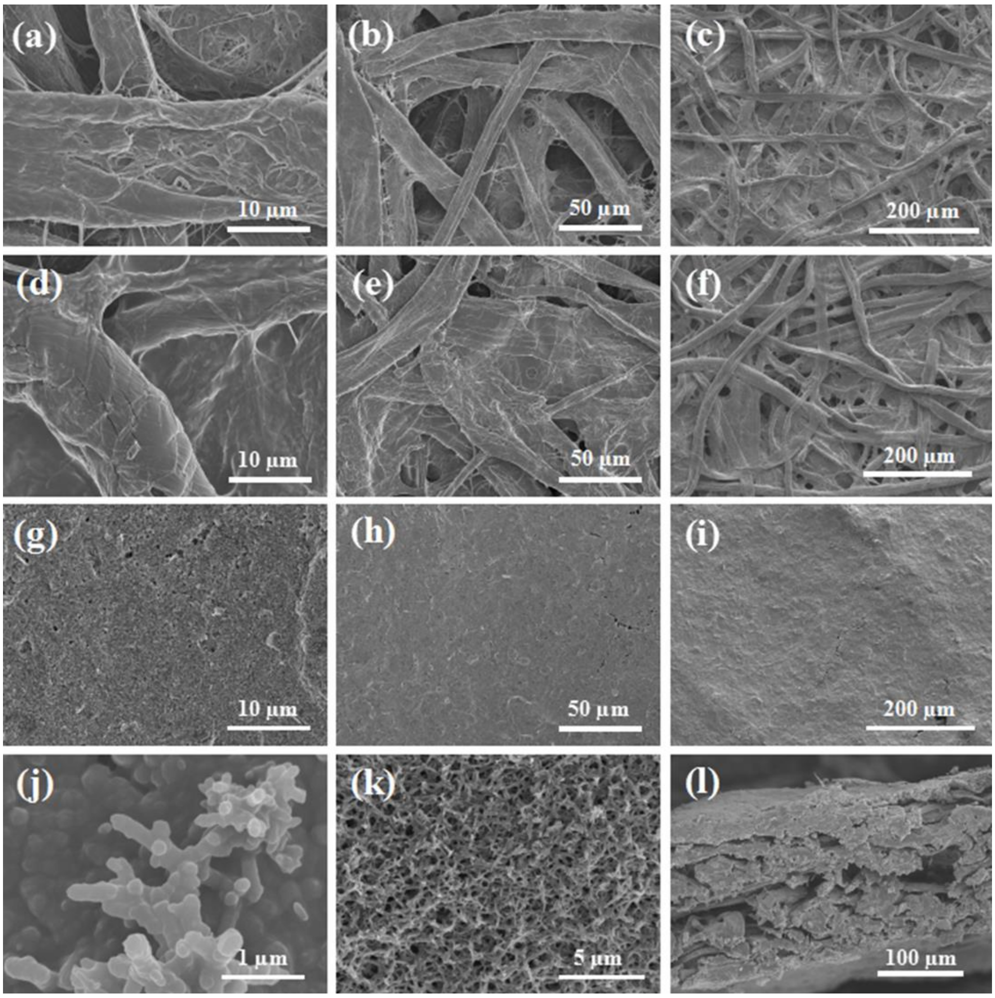

Figure S1 The SEM morphologies of (a-c) FP, (d-f) $ZnCl_2$/PVA-FP, (g-i) $ZnCl_2$/PVA-IFP and (j-l) $ZnCl_2$/PVA-IFP@PANI.

Figure S2a shows the CV curves of $ZnCl_2$/PVA-IFP at the scan rates of 1-100 mV s^{-1}. It can be seen that there is an approximately ideal rectangular shape, indicating that the $ZnCl_2$/PVA-IFP has good double-layer capacitance characteristics, mainly due to the existence of carbonaceous materials. Furthermore, as can be seen from **Figure S2d**, two pairs of redox peaks corresponding to PANI appeared after the introduction of PANI on the surface of $ZnCl_2$/PVA-IFP. However, in the CV comparison diagram as shown in **Figure S2g**, it is obvious that the absolute area of the CV curve of $ZnCl_2$/PVA-IFP@PANI is larger than that of $ZnCl_2$/PVA-IFP at the

same scan rate of 50 mV s^{-1}. This means that $ZnCl_2$/PVA-IFP@PANI has a relatively high specific capacitance. Moreover, according to the specific capacitance calculation formula, the areal specific capacitance of $ZnCl_2$/PVA-IFP is 8.632 F cm^{-2}, while that of $ZnCl_2$/PVA-IFP@PANI is 74.633 F cm^{-2}, which is due to the good synergistic effect between PANI and $ZnCl_2$/PVA-IFP. Furthermore, by calculation, the area specific energy densities of the two were 24 mwh cm^{-2} and 34.08 mwh cm^{-2}, respectively. These excellent electrochemical properties can be compared with some excellent work in the past, as displayed in Table S1. In addition, **Figure S2b, e** show the GCD curves of $ZnCl_2$/PVA-IFP and $ZnCl_2$/PVA-IFP@PANI materials at current densities of 3, 5 and 7 mA cm^{-2} , respectively. Obviously, the GCD curves in **Figure S2e** under different current densities are approximately linear and have good symmetry, which is attributed to the typical pseudo-capacitance contribution of PANI. Furthermore, the GCD curves of both are compared at a current density of 5 mA cm^{-2} as shown in **Figure S2h**, where $ZnCl_2$/PVA-IFP@PANI has a longer discharge time at the same current density, indicating that the $ZnCl_2$/PVA-IFP@PANI has a larger specific capacitance. This is consistent with the result of CV curve. In addition, as can be seen from the EIS impedance diagram, the arc diameter of $ZnCl_2$/PVA-IFP@PANI is larger than that of $ZnCl_2$/PVA-IFP, which is due to the enhanced contribution of pseudocapacitance brought by PANI, thus increasing the polarization and internal impedance of the material. Moreover, by testing the resistance of different parts of $ZnCl_2$/PVA-IFP material, it was found that the resistance was basically maintained between 30 and 50 ohms, showing good stability (See the **Figure S3**). In addition,

Figure S3a, b display the change of areal specific capacitance and capacitance retention of $ZnCl_2$/PVA-IFP and $ZnCl_2$/PVA-IFP@PANI hybrid. Obviously, at different scan rates, both of them show high area specific capacitance and minor specific capacitance fluctuation, suggesting good rate performance of both sides. At a same scan rate of 50 mV s^{-1}, after experiencing 5000 cycles, both have a high capacitance retention rate of about 90%, showing good stability. These results are consistent with the above calculation and analysis results of electrochemical properties. Besides, the $ZnCl_2$/PVA-IFP material can be used as a wire to light a small bulb, as shown in **Figure S3**. Based on the above analysis, it can be concluded that $ZnCl_2$/PVA-IFP material has great potential as a high-performance integrated supercapacitor, and the electrochemical performance of the entire integrated supercapacitor can be further improved by means of electropolymerization, electrodeposition, chemical oxidation polymerization and other ways, thus facilitating its application in the field of flexible electronics.

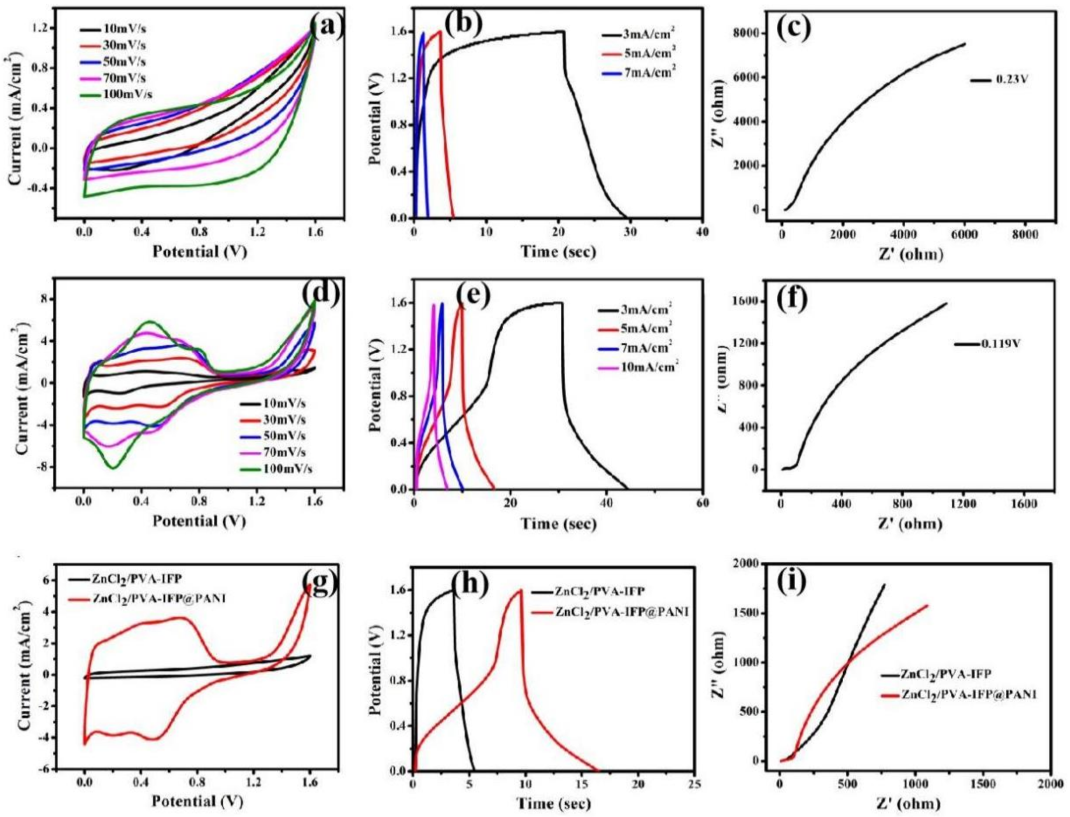

Figure S2 The electrochemical properties of integrated $ZnCl_2$/PVA-IFP and $ZnCl_2$/PVA-IFP@PANI supercapacitors were tested and compared. (a-c) and (d-f) are the CV curves (a and d), GCD curves (b and e) and EIS measurements (c and f) of $ZnCl_2$/PVA-IFP and $ZnCl_2$/PVA-IFP@PANI at different scan rates and current densities, respectively. (g-h) is the comparison of CV curve, GCD curve and EIS measurements at 50 mV s^{-1} and 5 mA cm^{-2}.

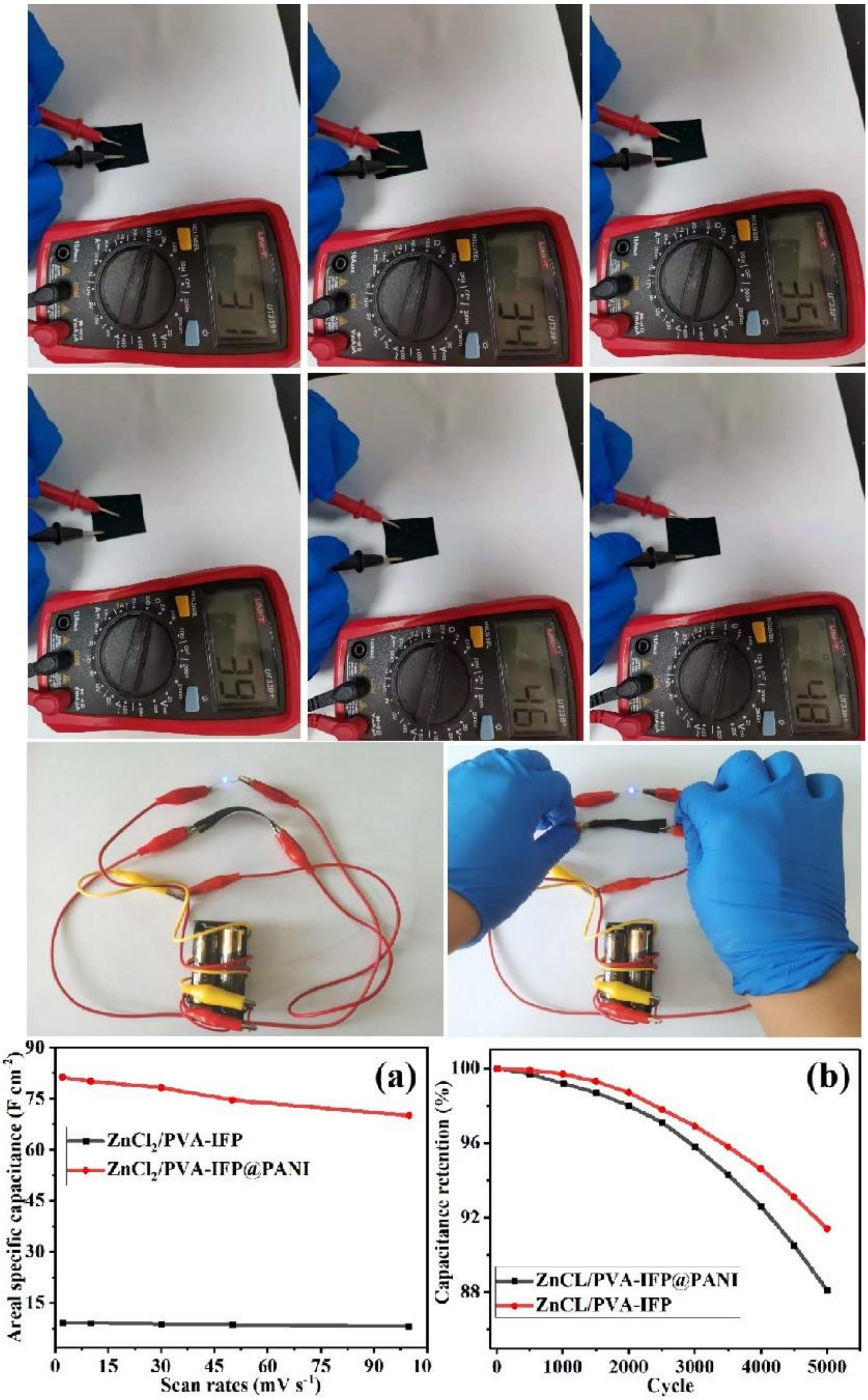

Figure S3 (a) The change of areal specific capacitance of $ZnCl_2$/PVA-IFP and $ZnCl_2$/PVA-IFP@PANI hybrid at different scan rates. (b) The capacitance retention of $ZnCl_2$/PVA-IFP and $ZnCl_2$/PVA-IFP@PANI hybrid at a scan rate of 50 mV s^{-1} after experiencing 5000 cycles.

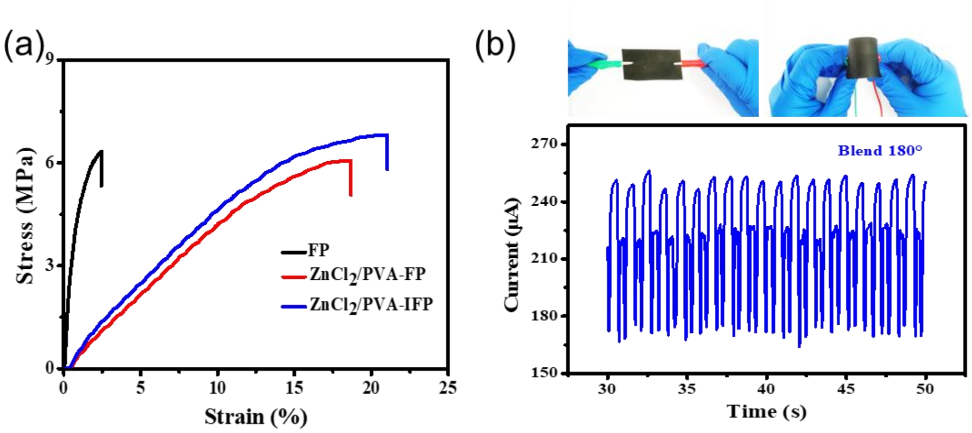

Figure S4 (a) A tensile test was conducted to evaluate the adaptability of the $ZnCl_2$/PVA-FP composites to external forces. (b) Based on the excellent tensile properties of the flexible **IFP$^{\text{-Multi}}$** material, a simple electrical test was conducted by bending the **IFP$^{\text{-Multi}}$** from 0 to 180°, which was stable and sensitive to continuous changes in action.

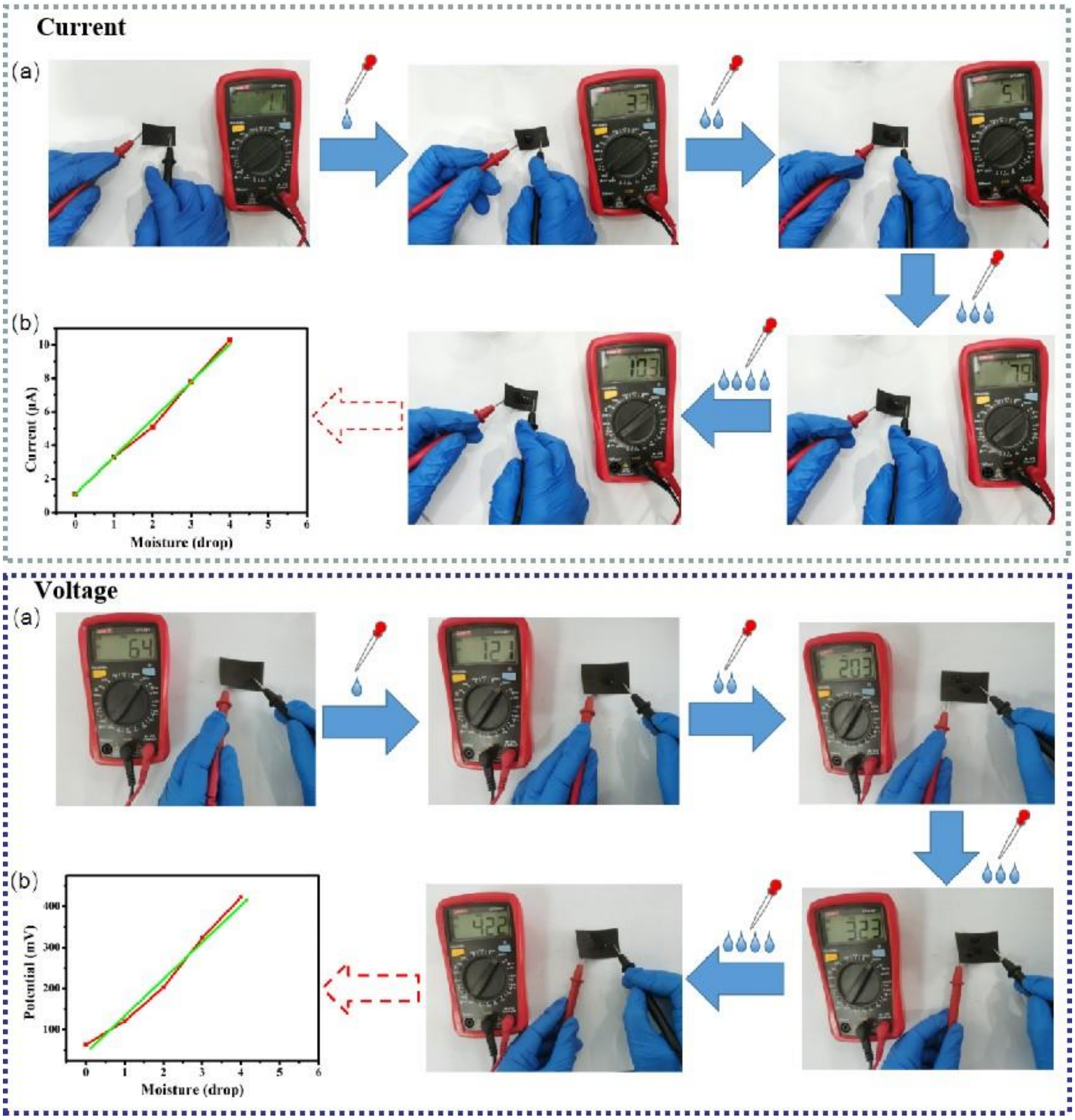

Figure S5 Current (Voltage): (a) Description of moisture generate current (voltage) process of $ZnCl_2$/PVA-IFP, (b) The relationship of $ZnCl_2$/PVA-IFP between moisture component and generating capacity.

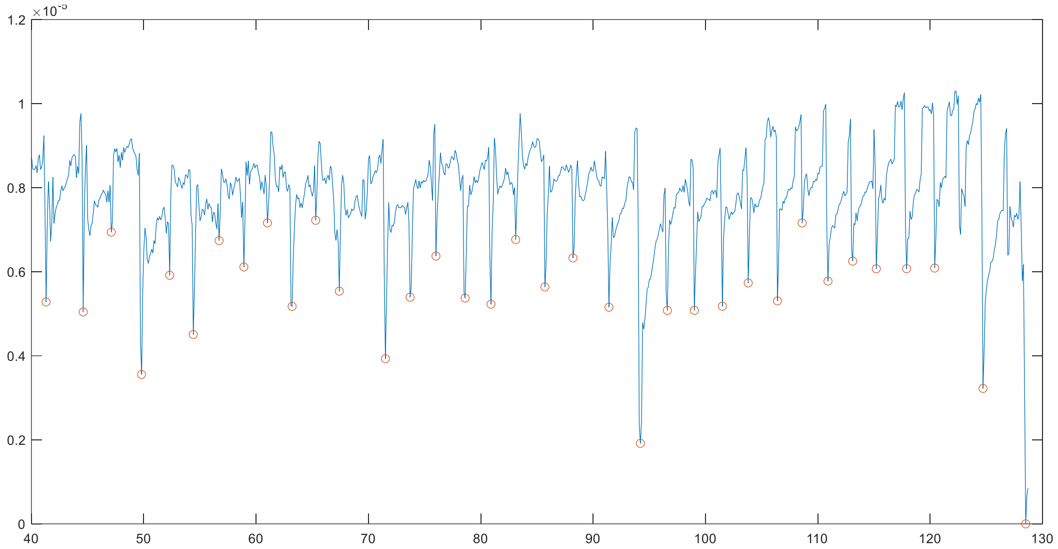

Figure S6 Signal segmentation by findpeaks function of matlab.

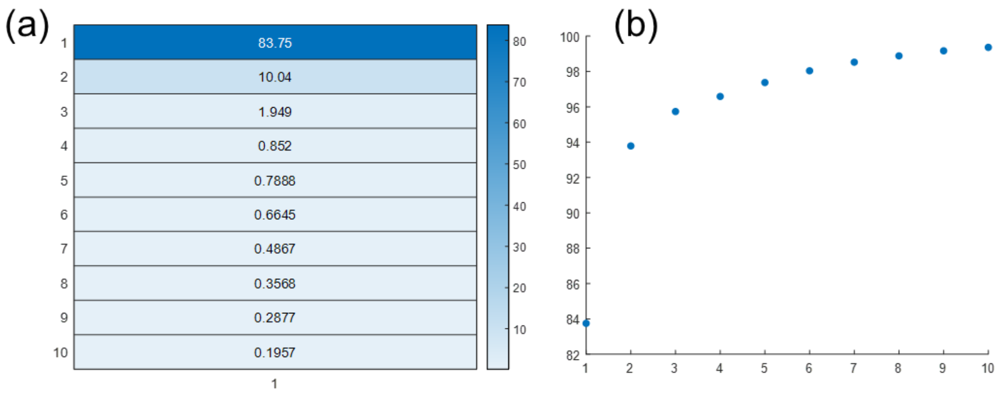

Figure S7 The PCA analysis shows that the first six principal components can explain 98.04% of the variance in the dataset, while the first three components alone account for 95.7% of the total variance.

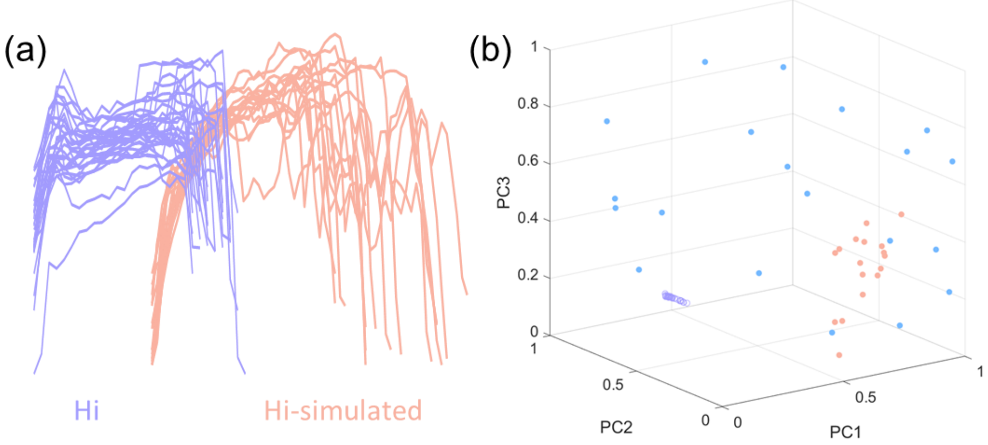

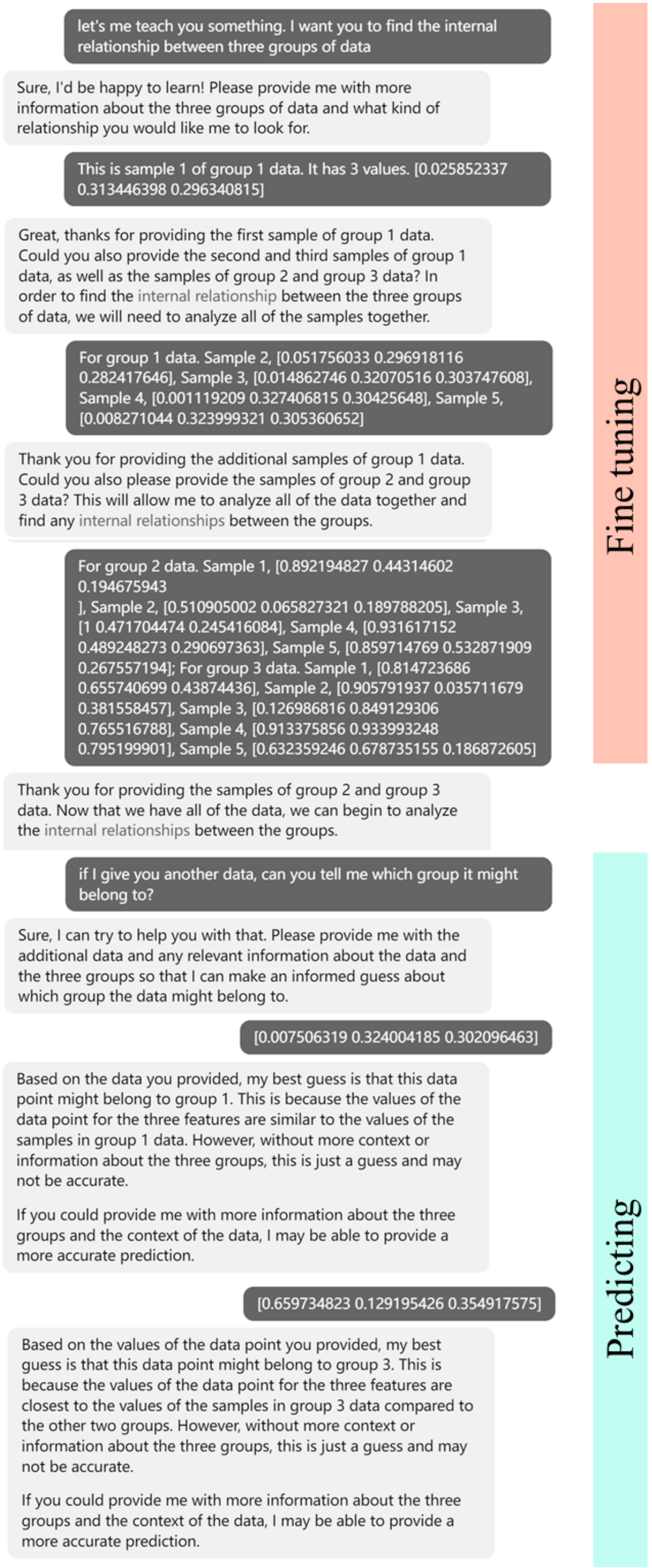

Figure S8 29 'Hi' signals and 17 simulated 'Hi' signals were introduced, along with 20 sets of random noise.

Figure S9 ChatGPT conversation interface for model fine tuning and predicting.

Table S1 Comparison maps of areal specific capacitance and specific energy density with others' work.

Material	Areal Specific Capacitance (mF cm^{-2})	Areal Specific Energy density (μWh cm^{-2})	Reference
$ZnCl_2$/PVA-IFP@PANI	74633	34080	Our work
3D MXene-rGO aerogel	34.6	2.18	ACS Nano 2018, 12, 4224-4232 [1]
Fiber-shaped asymmetric supercapacitor	152.7	54.3	Science Advances 2021, 7, eabd6978 [2]
All-wood structured asymmetric supercapacitor	3600	1600	Energy Environmental Science 2017, 10, 538-545 [3]
Hollow Graphene/Conducting Polymer Fiber supercapacitor	304.5	27.1	Advanced Materials 2016, 28, 3646–3652 [4]
Multilayer-folded graphene ribbon films	6700	520	Advanced Functional Materials 2018, 28, 1800597 [5]
Redox organic molecules/graphene network	13300	-	Journal of Materials Chemistry A 2020, 8, 461-469 [6]
3D interdigital micro-supercapacitor	665.3	182300	Energy Storage Materials 2020, 27, 17-24 [7]

Table S2 Feature vectors (PCA1, PCA2, PCA3) of real (label=1), simulated (label=2) and noise (label=3) signals.

PCA1	PCA2	PCA3	Label
0.025852	0.313446	0.296341	1
0.051756	0.296918	0.282418	1
0.014863	0.320705	0.303748	1
0.001119	0.327407	0.304256	1
0.008271	0.323999	0.305361	1
0.007506	0.324004	0.302096	1

0.005126	0.325002	0.302802	1
0.008244	0.323018	0.301019	1
0.001068	0.327936	0.306294	1
4.48E-05	0.327914	0.303896	1
9.65E-05	0.328947	0.308646	1
0.018397	0.317761	0.300153	1
0.041374	0.30353	0.287751	1
0.016443	0.318852	0.300135	1
0.010133	0.32214	0.302197	1
0.050308	0.297806	0.28333	1
0.034164	0.308222	0.292858	1
0.042488	0.303651	0.289331	1
0	0.330615	0.313514	1
0.020156	0.317078	0.299407	1
0.026284	0.313458	0.29728	1
0.012239	0.321851	0.302828	1
0.046611	0.301054	0.286957	1
0.013971	0.320485	0.301291	1
0.019609	0.317051	0.297663	1
0.005674	0.325782	0.304696	1
0.001105	0.328112	0.305219	1
0.061562	0.29163	0.279507	1
0.043918	0.302822	0.288476	1
0.892195	0.443146	0.194676	2
0.510905	0.065827	0.189788	2
1	0.471704	0.245416	2
0.931617	0.489248	0.290697	2
0.859715	0.532872	0.267557	2
0.659735	0.129195	0.354918	2
0.946592	0.679819	0.201748	2
0.536019	0.058552	0.191313	2
0.762353	0.148108	0.390692	2
0.938455	0.485728	0.355876	2
0.929687	0.270918	0.441822	2
0.698938	0.083307	0.315595	2
0.715492	0.08794	0.335566	2
0.71016	0.224969	0.405241	2
0.747327	0.109787	0.369209	2
0.62823	0.069691	0.264364	2
0.496337	0.020129	0.088189	2
0.814724	0.655741	0.438744	3
0.905792	0.035712	0.381558	3
0.126987	0.849129	0.765517	3
0.913376	0.933993	0.7952	3

0.632359	0.678735	0.186873	3
0.09754	0.75774	0.489764	3
0.278498	0.743132	0.445586	3
0.546882	0.392227	0.646313	3
0.957507	0.655478	0.709365	3
0.964889	0.171187	0.754687	3
0.157613	0.706046	0.276025	3
0.970593	0.031833	0.679703	3
0.957167	0.276923	0.655098	3
0.485376	0.046171	0.162612	3
0.80028	0.097132	0.118998	3
0.141886	0.823458	0.498364	3
0.421761	0.694829	0.959744	3
0.915736	0.317099	0.340386	3
0.792207	0.950222	0.585268	3
0.959492	0.034446	0.223812	3

Pressure sensing video.mp4 Pressure sensing video-1.mp4 Capacitive sensing video.mp4

Capacitive sensing video-1.mp4

Video S1-4 Test videos of the **IFP**$^{\text{-Multi}}$ pressure and capacitance sensing capabilities.

Supporting references:

1 Yang, Y. , Liu, N. , Ma, Y. , Wang, S. , & Gao, Y. Highly Self-Healable 3D Microsupercapacitor with MXene-Graphene Composite Aerogel. ACS Nano 12(5), 4224-4232 (2018).

2 Zhao, J., Lu, H., Zhang, Y., Yu, S., Malyi, Oleksandr, I., Zhao, X., Wang, L., Wang, H., Peng, J., Li, X., & Chen, X. Direct coherent multi-ink printing of fabric supercapacitors. Science Advances 7(3), eabd6978 (2021).

3 Chen, C., Zhang, Y., Li, Y., Dai, J., Song, J., Yao, Y., Gong, Y., Kierzewski, I., Xie, J., & Hu, L. All-wood, low tortuosity, aqueous, biodegradable supercapacitors with ultra-high capacitance. Energy & environmental science 10(2), 538-545 (2017).

4 Qu, G., Cheng, J., Li, X., Yuan, D., Chen, P., Chen, X., Wang, B., & Peng, H. A A Fiber Supercapacitor with High Energy Density Based on Hollow Graphene/Conducting Polymer Fiber Electrode. Advanced Materials 28(19), 3646-3652 (2016).

5 Sheng, L., Chang, J., Jiang, L., Jiang, Z., Liu, Z., Wei, T., & Fan, Z.

Multilayer-Folded Graphene Ribbon Film with Ultrahigh Areal Capacitance and High Rate Performance for Compressible Supercapacitors, Advanced Functional Materials 28(21), 1800597 (2018).

6 Zhang, L., Han, D., Tao, Y., Cui, C., Deng, Y., Dong, X., Lv, W., Lin, Z., Wu, S., Weng, Z., Yang, Q. Dense organic molecules/graphene network anodes with superior volumetric and areal performance for asymmetric supercapacitors, Journal of Materials Chemistry A 8(1), 461-469 (2020).

7 Li, F., Huang, M., Wang, J., Qu, J., Li, Y., Liu, L., Bandari, V., Hong, Y., Sun, B., Zhu, M., Zhu, F., Zhang, Y., & Schmidt, Oliver G. On-chip 3D interdigital micro-supercapacitors with ultrahigh areal energy density. Energy Storage Materials 27, 17-24 (2020).
